# Repurposing Bedaquiline for Effective Non-Small Cell Lung Cancer (NSCLC) Therapy as Inhalable Cyclodextrin-Based Molecular Inclusion Complexes

**DOI:** 10.3390/ijms22094783

**Published:** 2021-04-30

**Authors:** Vineela Parvathaneni, Rasha S. Elbatanony, Mimansa Goyal, Tejashri Chavan, Nathan Vega, Srikanth Kolluru, Aaron Muth, Vivek Gupta, Nitesh K. Kunda

**Affiliations:** 1 Department of Pharmaceutical Sciences, College of Pharmacy and Health Sciences, St. John’s University, Queens, NY 11439, USA; vineela.parvathaneni16@my.stjohns.edu (V.P.); elbatanr@stjohns.edu (R.S.E.); mimansa.goyal18@my.stjohns.edu (M.G.); tejashri.chavan18@stjohns.edu (T.C.); mutha@stjohns.edu (A.M.); 2 Department of Pharmaceutical Technology, Faculty of Pharmaceutical Sciences and Pharmaceutical Industries, Future University in Egypt, Cairo 11835, Egypt; 3 School of Pharmacy and Health Sciences, Keck Graduate Institute, 535 Watson Dr, Claremont, CA 91711, USA; nvega18@students.kgi.edu (N.V.); srikanth_kolluru@kgi.edu (S.K.)

**Keywords:** bedaquiline, sulfobutylether-β-cyclodextrin, non-small cell lung cancer, inhalation, molecular docking

## Abstract

There is growing evidence that repurposed drugs demonstrate excellent efficacy against many cancers, while facilitating accelerated drug development process. In this study, bedaquiline (BDQ), an FDA approved anti-mycobacterial agent, was repurposed and an inhalable cyclodextrin complex formulation was developed to explore its anti-cancer activity in non-small cell lung cancer (NSCLC). A sulfobutyl ether derivative of β-cyclodextrin (SBE-β-CD) was selected based on phase solubility studies and molecular modeling to prepare an inclusion complex of BDQ and cyclodextrin. Aqueous solubility of BDQ was increased by 2.8 × 10^3^-fold after complexation with SBE-β-CD, as compared to its intrinsic solubility. Solid-state characterization studies confirmed the successful incorporation of BDQ in the SBE-β-CD cavity. In vitro lung deposition study results demonstrated excellent inhalable properties (mass median aerodynamic diameter: 2.9 ± 0.6 µm (<5 µm) and fine particle fraction: 83.3 ± 3.8%) of BDQ-CD complex. Accelerated stability studies showed BDQ-CD complex to be stable up to 3 weeks. From cytotoxicity studies, a slight enhancement in the anti-cancer efficacy was observed with BDQ-cyclodextrin complex, compared to BDQ alone in H1299 cell line. The IC_50_ values for BDQ and BDQ-CD complex were found to be ~40 µM in case of H1299 cell line at 72 h, whereas BDQ/BDQ-CD were not found to be cytotoxic up to concentrations of 50 µM in A549 cell line. Taken together, BDQ-CD complex offers a promising inhalation strategy with efficient lung deposition and cytotoxicity for NSCLC treatment.

## 1. Introduction

Non-small cell lung cancer (NSCLC) is the leading cause of cancer deaths worldwide and has drawn significant attention from researchers in the field [1]. With increased resistance to conventional anti-cancer therapeutics and severe systemic toxicities observed with newer treatment modalities, there is a dire need to discover and develop new therapeutics and delivery systems to achieve safe and efficacious medications [2]. To overcome this emerging challenge, repurposing of old drugs has gained much traction and has led to the identification of some promising FDA-approved drugs for lung cancer treatment [3,4].

Quinoline derivatives have many diverse applications as anti-malarial, anti-viral, anti-bacterial, anti-asthmatic, etc., agents [5,6]. In addition, quinoline scaffold and several quinoline analogs have been reported as anti-cancer agents with promising anti-proliferative activity against multiple cancer cell lines, including HeLa (cervical cancer cell line) and MDA-MB-435 (melanoma) [7]. In particular, diaryl quinolines demonstrating cytotoxic activity against different cancer types lead to the identification of anti-cancer efficacy of bedaquiline (BDQ) [8,9]. 

BDQ is an FDA-approved anti-mycobacterial agent used in the treatment of multi-drug resistant pulmonary tuberculosis (TB). BDQ acts through inhibition of the bacterial ATP-synthase and is often used in combination with other antibiotics and has demonstrated activity against both drug-sensitive and drug-resistant TB [10]. Recent studies have reported BDQ’s ability to inhibit mitochondrial respiration and glycolysis in lung cancer cells, resulting in cancer cell growth inhibition and angiogenesis inhibition, thereby suppressing the tumor growth [9,10]. While bedaquiline demonstrated anti-cancer efficacy against lung cancer, major limitations are associated with its physicochemical properties and efficient delivery. BDQ belongs to Biopharmaceutics Classification System class II, i.e., the drug has low water solubility and high permeability. The low solubility of BDQ results in poor bioavailability and efficacy, and requires administration of high doses for a long-term. Such dosage regimens often result in therapeutic outcomes with severe systemic side effects. To overcome these challenges, we developed bedaquiline-cyclodextrin inclusion complex to improve aqueous solubility of BDQ which further assists in achieving enhanced anti-tumoral effectiveness.

Cyclodextrins are extensively used to improve the water-solubility of poorly water-soluble drugs. CD is reported as a safe excipient via oral, intravenous, and inhalation administration [11,12]. Beta-cyclodextrins (β-CDs) are the most frequently used cyclodextrins in pharmaceutical formulations, due to their ease of availability, cost effectiveness, and ability to readily form inclusion complexes with poorly water-soluble drugs [13,14]. In particular, sulfobutyl ether-β-CD (SBE-β-CD) has been used by several groups, including ours, to enhance the aqueous solubility of lipophilic drugs [15,16,17]. Moreover, SBE-β-CD is suggested to be safer than other derivatives of cyclodextrins [18,19]. 

Pulmonary delivery allows for non-invasive administration of drugs directly to the deep lung regions providing high drug concentrations, compared to systemic delivery at the same dose [20,21]. Accumulation into the deep lungs is imperative for treatment of NSCLC [22,23]. Especially, accumulation of drug-cyclodextrin complexes in the lungs after pulmonary delivery has been studied by several researchers and an improved efficacy through CD complexation in NSCLC has been reported earlier [24,25,26,27]. To date, there have been no studies on the inclusion complexation of BDQ using cyclodextrins. Moreover, reports on the pulmonary delivery of cyclodextrin inclusion complex of BDQ are not available so far. Therefore, we evaluated the aerodynamic performance of BDQ-cyclodextrin complex, an entirely novel domain for BDQ delivery. 

In this study, we hypothesize that the BDQ-CD inclusion complex improves solubility of BDQ and enhances its anti-cancer efficacy in NSCLC cell lines. The overall aim of this study was to establish an efficacious formulation strategy (complexation with CD) to enhance biological efficacy, while overcoming the solubility issues associated with BDQ.

## 2. Results and Discussion

### 2.1. UV Method Development for Bedaquiline (BDQ)

**Figure 1:**
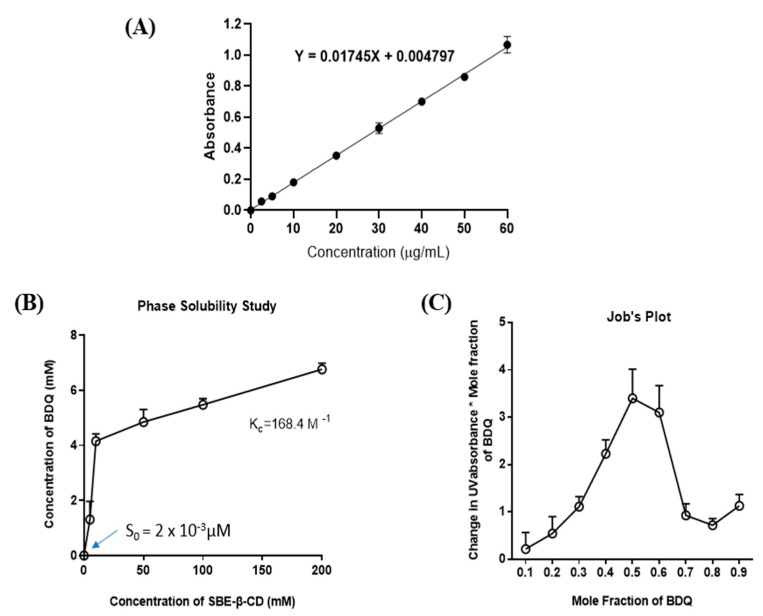
(**A**) Calibration curve for bedaquiline from the standard solutions ranging from 0–60 µg/mL was plotted by using UV-1600 PC Spectrophotometer at 285 nm. Data represent mean ± SD (*n* = 3). (**B**) Phase solubility diagram of bedaquiline in water with Sulfobutyl Ether β-Cyclodextrin (SBE-β-CD). (**C**) Job’s continuous variation plot of bedaquiline-CD complex. Results indicated 1:1 stoichiometry during the formation of inclusion complex. All data represent mean ± SD (*n* = 3).

A UV spectrophotometric method was developed for determining BDQ concentration in analytical samples. All the standard solutions were scanned in the UV range 200–600 nm and BDQ showed maximum absorbance at 285 nm. A calibration curve was plotted for BDQ concentration (0–60 µg/mL) and absorbance. Linear regression data for BDQ calibration curve showed a good linear relationship over the concentration range 0–60 µg/mL tested, as represented in Figure 1A. This developed method exhibited excellent linearity (R^2^ = 0.99) with a linear regression equation:

*
y 
*
= 0.01745X + 0.004797 (1)

### 2.2. Phase-Solubility Studies

In this study, complexes of BDQ with SBE-βCD were prepared and the impact on aqueous solubility of BDQ was evaluated. In many of our recent studies, we have reported SBE-β-CD to enhance the solubility and stability significantly, compared to other CD types [17,25,28,29]. As shown in Figure 1B, the solubility of BDQ linearly increased with increasing concentration of SBE-β-CD in water, which indicates that the phase solubility curve represents an A_L_-type system [30]. The A_L_-type phase diagram also revealed a 1:1 stoichiometry between BDQ and SBE-β-CD during the formation of inclusion complexes, which indicated that one molecule of drug forms a complex with one molecule of CD. This observation was also supported by the linear phase solubility diagram, wherein the slope value was <1 indicating the formation of complexation at a 1:1 molar ratio of BDQ and SBE-β-CD [29]. Phase solubility diagrams were used to quantify the solubilization capabilities of SBE-β-CD. Intrinsic solubility of BDQ was found to be 2.400 ± 0.002 μM at 25 °C. With the inclusion of SBE-β-CD, the resultant complex increased the solubility of BDQ to 6761.1 ± 0.2 μM (a ≈2.8 × 10^3^-fold solubility enhancement). To further understand the complexation mechanism between BDQ and cyclodextrin SBE-β-CD, the stability rate constant (K_c_) was calculated using the slope and intercept values (S_0_) from phase solubility data. S_o_ value (2 × 10^−3^ µM) observed at 0 mM concentration of SBE-β-CD has been used in the K_c_ calculation. The K_c_ value was found to be 168.4 M^−1^, which was in the optimum range of 100 to 1000 M^−1^ indicating that the complex formed was stable [28]. A stable complex ensures stronger interactions between the guest molecules (drug) and host molecules (SBE-β-CD) that may improve the solubility and therapeutic activity of the drug [28]. In addition, the complexation efficiency (CE) was found to be 0.4 (reported average for cyclodextrin complexes is 0.3), indicating that on average, only about one out of every three cyclodextrin molecules present in a given complexation medium is in the complex for hydrophobic drug molecules [25]. Angiolini et al. prepared cyclodextrin inclusion complexes of rifampicin (anti-tuberculosis agent) and observed an improvement in aqueous solubility by 23-fold when using methylated cyclodextrin, heptakis (2,6-diO-methyl)-β-cyclodextrin [31]. Salzano et al. prepared cyclodextrin nanocarrier system for two synergistic drugs ethionamide and its booster molecule, BDM43266. By complexation with β-cyclodextrin, the authors increased the aqueous solubility of ethionamide and booster molecule 10 and 90 fold, respectively [32]. In an earlier study published by our group, a 66-fold increase in solubility was reported for resveratrol after complexation with SBE-β-CD [25]. In the current study, a superior solubility enhancement (2800-fold) of BDQ was observed after complexation signifying the inclusion complex formation.

### 2.3. Continuous Variation Method (Job’s Plot)

An alteration in the UV spectra of a drug molecule is observed after the formation of inclusion complexes, due to modified solvent microenvironment during complexation. This study involves preparing a series of solutions containing both the host (SBE-β-CD) and the guest (BDQ) in varying proportions so that a complete range of mole ratios is sampled and where the total concentration of drug + SBE-β-CD is kept constant for each solution. The experimentally observed parameter is a host or guest chemical shift that is sensitive to complex formation. The molar ratio, at which a maximum shift in the UV absorbance of drug occurs, is considered the stoichiometric ratio. The results of the Job’s plot analysis for BDQ-CD inclusion complex showed a maximum change (UV absorbance × mole fraction change) was obtained at a mole fraction value 0.5, *indicating* a *1:1 stoichiometric* ratio for the inclusion complex (Figure 1C). A similar type of stoichiometry was also observed from the phase solubility diagram. Therefore, BDQ and SBE-β-CD were used in a 1:1 molar ratio for formation of the inclusion complex for further characterization and therapeutic evaluation. Job’s plot is widely used for applications in pharmacology, biomedicine, microbiology, forensic science, and allied fields, due to the simplicity of theoretical foundation and straightforward experimental application. It is based on the study of a graphical representation of analytical signal versus ligand molar fraction. This method is widely reported in the literature, primarily to report estimation of stoichiometry of compounds [33]. Some of the contributions of the Job’s plot analysis in the pharmaceutical field are briefly discussed here. In a previous study, we formed an inclusion complex of afatinib and sulfobutylether β-cyclodextrin and analyzed the stoichiometric ratio of the components in the complex using the Job’s plot. The results showed a 1:1 molar ratio similar to the current investigation [29]. Vaidya et al also prepared a sulfonated β-cyclodextrin (Sβ-CD)-nintedanib inclusion complex, and analysis using the Job’s plot indicated a stoichiometric ratio of 1:1 for the inclusion complex formed [17]. Overall, simplicity and capability of the Job’s plot enables researchers to investigate thoroughly and obtain insights into the stoichiometries underlying association of different molecules.

### 2.4. Solid State Characterization of BDQ-SBE-β-CD Complex

#### 2.4.1. ^1^H-Nuclear Magnetic Resonance Spectroscopy (^1^H-NMR)

**Figure 2:**
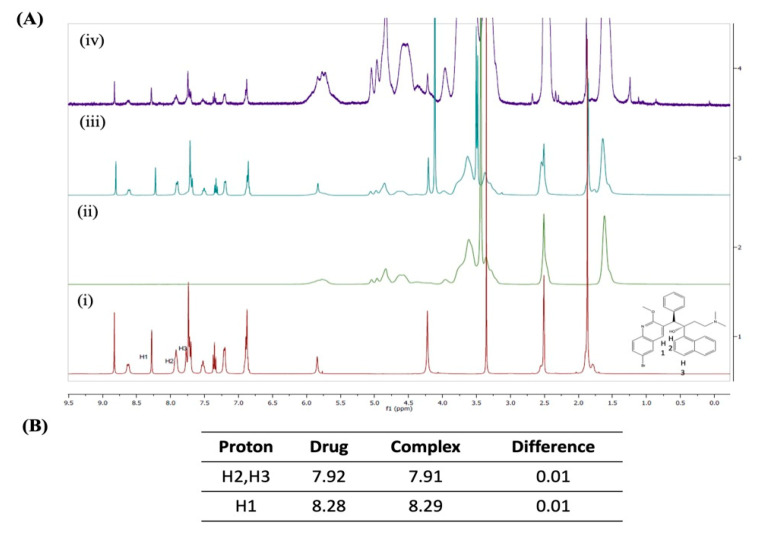
(**A**) NMR spectra of bedaquiline (i), SBE-β-CD (ii), the physical mixture of bedaquiline and SBE-β-CD (iii), and the inclusion complex BDQ-CD (iv). (**B**) Summary table for NMR chemical shift values.

Figure 2A shows the ^1^H NMR chemical shifts of BDQ (i), SBE-β-CD (ii), the physical mixture of BDQ and SBE-β-CD (iii), and the BDQ-CD inclusion complex (iv). As shown in Figure 2A, all of the proton chemical shifts observed for the drug alone were very similar to those observed for the physical mixture and inclusion complex (<0.01 ppm change) except for H1, H2, and H3. Notably, larger chemical shifts were observed for aromatic protons H1, H2, and H3, which exhibited a modest change of 0.01 ppm, as represented in Figure 2B. These chemical shift changes suggest that this aspect of the BDQ scaffold finds itself within the cavity of cyclodextrin inclusion complex. Nevertheless, a distinct proton shifting was not observed with NMR study. Hence, other analytical studies, such as XRD and DSC were employed to ascertain the formation of internal interaction between BDQ and CD, as reported earlier [34].

#### 2.4.2. FT-IR Analysis

**Figure 3:**
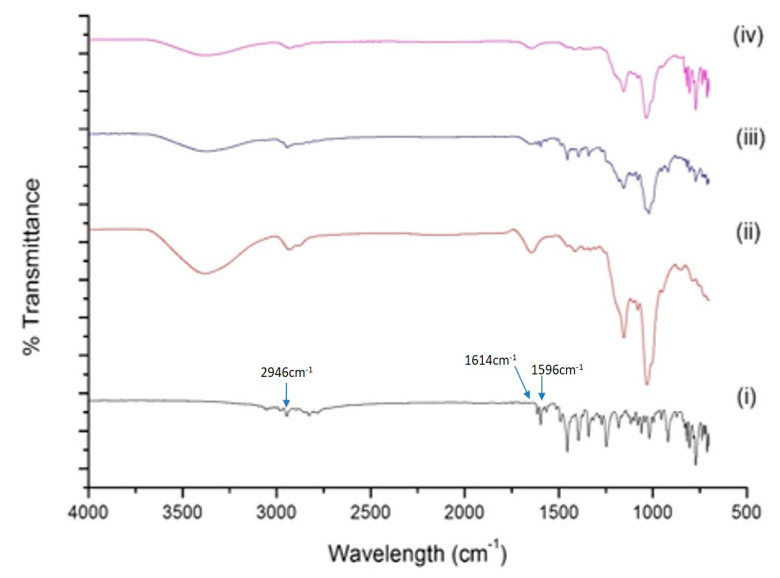
FT-IR spectra of bedaquiline (i), SBE-β-CD (ii), the physical mixture of bedaquiline and SBE-β-CD (iii), and the inclusion complex BDQ-CD (iv).

Inclusion of BDQ in SBE-βCD results in interaction between functional groups of drug and CD molecules, which can be estimated using FT-IR. The FT-IR spectra of BDQ, SBE-β-CD, the physical mixture of BDQ and SBE-β-CD, and the BDQ-CD complex are presented in Figure 3. The spectra of BDQ (Figure 3 (i)) showed major peaks for ether (1180 cm^−1^ and 1081 cm^−1^), alcohol (3178 cm^−1^), and aromatic (3028 cm^−1^, 3054 cm^−1^, 3028 cm^−1^, 2975 cm^−1^, 2946 cm^−1^, 1614 cm^−1^, 1596 cm^−1^, and 1455 cm^−1^) functional groups, similar to a previous report [35]. The FT-IR spectrum of SBE-β-CD (Figure 3 (ii)) alone exhibited characteristic peaks at about 3380 cm^−1^ for O-H stretching, 2930 cm^−1^ for C-H stretching, and 1034 cm^−1^ for C-O stretching vibration. The spectrum of physical mixture (Figure 3 (iii)) of BDQ and SBE-β-CD demonstrated a super-positioned spectrum of both compounds, but with less intense absorption peaks of BDQ at around 1020, 1341, 1596, and 1614 cm^−1^, demonstrating a weak interaction. The FT-IR spectrum of the BDQ-CD inclusion complex (Figure 3 (iv)) showed no distinct characteristic aromatic peaks of BDQ 2946 cm^−1^, 1596 cm^−1^, and 1614 cm^−1^. The band of -OH group of SBE-β-CD at 3380 cm^−1^ was also observed to be smoothened, which may be due to involvement in intermolecular hydrogen bonding with BDQ molecules during complexation, thus indicating BDQ’s successful encapsulation inside the hydrophobic cavity of SBE-β-CD molecule. These results provide an insight about incorporation of several functional groups of BDQ in the cavity of SBE-β-CD molecule during inclusion process. This was similar to a phenomenon observed by Momin et al., in which the hydroxyl band of pure β-cyclodextrin was narrowed in the FT-IR spectrum of inclusion complex compared to plain drug and was attributed to formation of the inclusion complex [35]. FTIR has been used for the characterization of cyclodextrin inclusion complex, as reported in several studies [28,29,36]. For instance, inclusion of afatinib into SBE-β-CD have been confirmed by Parvathaneni et al. [29]. In another study, an inclusion complex of azomethine-β-CD has been characterized by Sambasevam et al., using FTIR study [36].

#### 2.4.3. P-XRD Studies

**Figure 4:**
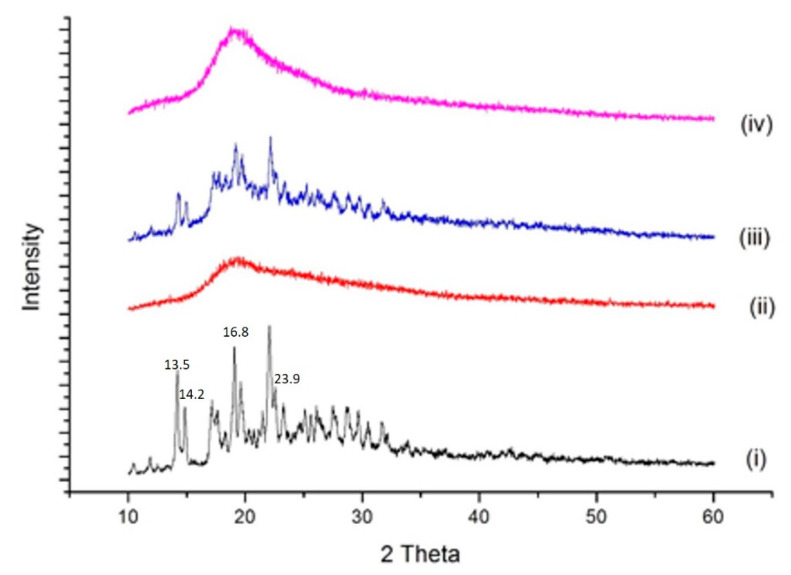
XRD spectra of bedaquiline (i), SBE-β-CD (ii), the physical mixture of bedaquiline and SBE-β-CD (iii), and the inclusion complex BDQ-CD (iv).

Powder X-ray diffraction (P-XRD) studies were performed to detect the pure and complexed drug. Results of diffractometry studies of BDQ, SBE-βCD, physical mixture, and BDQ-CD complex are displayed in Figure 4. As shown, BDQ (Figure 4 (i)) exhibited several sharp, intense characteristic peaks at diffraction angles of 10.4, 13.5, 14.2, 15.6, 16.8, and 23.9 illustrating the crystalline nature of BDQ, which is in agreement with literature [35]. However, due to the amorphous nature of SBE-β-CD (Figure 4 (ii)), its diffractogram did not show any sharp peaks. The physical mixture (Figure 4 (iii)) of BDQ and SBE-β-CD exhibited several intense peaks of BDQ at 2θ values of 10.3, 13.5, 14.3, 15.0, 16.8, and 23.4; but with less intensities, which may be attributed to interaction with SBE-β-CD. Furthermore, for the BDQ-CD inclusion complex (Figure 4 (iv)), the XRD spectra showed no sharp peaks for BDQ, which confirmed our previous observation about the incorporation of BDQ into the CD cavity during complexation. Moreover, the crystalline to amorphous change during the freeze-drying process may have also caused the disappearance of characteristic BDQ peaks [37,38]. Change of drug from crystalline to amorphous form after complexation is also indicative of increased molecular mobility and energy contributing to improved aqueous solubility, as observed for BDQ-CD complex [28].

#### 2.4.4. DSC Studies

**Figure 5:**
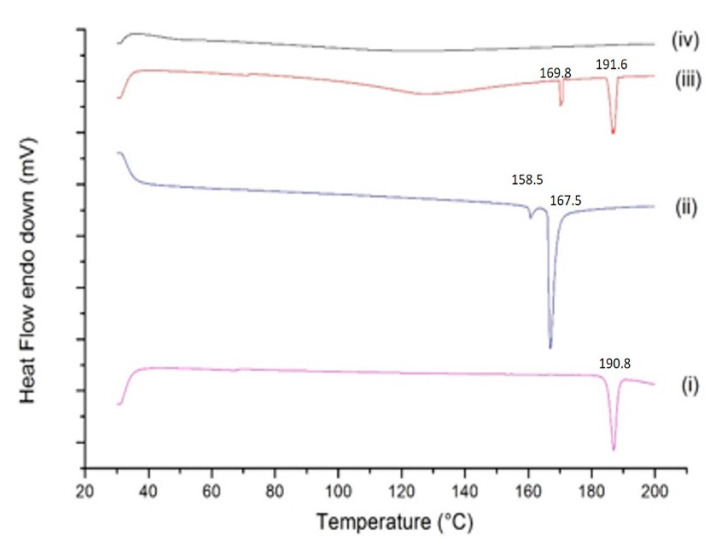
Thermograms of bedaquiline (i), SBE-β-CD (ii), the physical mixture of bedaquiline and SBE-β-CD (iii), and the inclusion complex BDQ-CD (iv).

DSC study was used to evaluate the thermal behavior of inclusion complex and pure drug, with successful complexation primarily indicated by absence of the drug peak. [39]. The thermogram of BDQ (Figure 5 (i)) demonstrates a sharp and intense endothermic peak at 190.8 °C, corresponding with its melting point and indicating the crystallinity of BDQ, which is in agreement with previous reports [40]. SBE-β-CD thermogram displayed two sharp endothermic peaks at 158.5 °C and 167.5 °C, as shown in Figure 5 (ii). Physical mixture of BDQ and SBE-β-CD, as observed in Figure 5 (iii), displayed the characteristic peaks of BDQ (at 191.6 °C) and endothermic peak at 169.8 °C of SBE-β-CD showing presence of both the components. However, physical mixture displayed slight shift in the peaks suggesting weak interaction between BDQ and SBE-β-CD, during the physical mixture preparation. The thermogram of the BDQ-CD inclusion complex shown in Figure 5 (iv) demonstrated an absence of characteristic sharp endothermic peaks of both BDQ and SBE-β-CD, suggestive of successful inclusion of BDQ into the SBE-β-CD cavity. Similar observations were presented in our recent study, where peaks referring to both drug and CD were absent in the drug-CD inclusion complex indicating the inclusion of drug inside the CD’s hydrophobic cavity [29]. The disappearance of the dehydration peak of CD can be explained by the fact that BDQ occupied the place of water inside the CD’s cavity, proving the formation of BDQ-CD inclusion complex as reported earlier [41]. These findings further provide support to XRD results indicating successful and complete inclusion of BDQ in the SBE-βCD cavity during complexation.

### 2.5. Accelerated Stability Study

**Figure 6:**
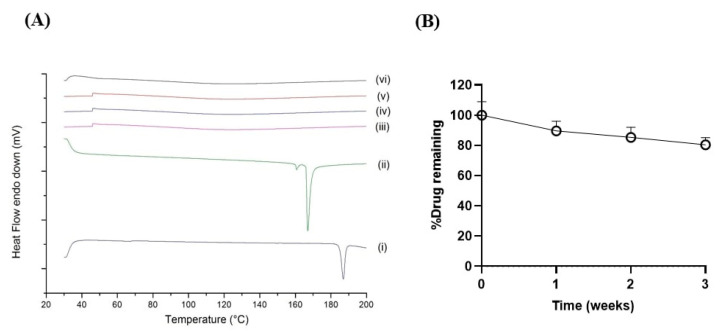
Accelerated stability testing at storage conditions (40 °C, 75%RH) (**A**) thermograms of bedaquiline (i), SBE-β-CD (ii) and BDQ-CD at week 0 (iii), 1 (iv), 2 (v) and 3 (vi). (**B**) Percentage drug remaining (%) in the CD inclusion complex versus time (weeks). Data represented as mean ± SD (*n* = 3).

In an accelerated stability test, the rate of degradation is accelerated under stressful conditions, including elevated temperature and humidity. The physicochemical properties of the samples, such as their crystallinity (P-XRD), thermal behavior (DSC), and percent drug content were evaluated over a period of exposure under these stress conditions [42]. In brief, BDQ and freeze dried BDQ-CD samples were stored for three weeks at accelerated conditions of temperature and humidity (40 ± 2 °C/75 ± 5% relative humidity). These conditions are the recommended accelerated storage conditions by the International Council for Harmonization of Technical Requirements for Pharmaceuticals for Human use (ICH) guidelines [42]. Solid state characterization studies (DSC: Figure 6A, to evaluate thermal behavior), and determination of % drug remaining (Figure 6B) in samples each week were performed for 3-weeks, and data were compared with that of fresh (week 0) BDQ-CD inclusion complex. These studies revealed the high stability of the BDQ-CD complex under accelerated stability conditions. DSC study (Figure 6A) demonstrated that amorphous state of the inclusion complex was retained after 3 weeks of storage, as characteristic, BDQ endotherms were not observed after analyzing the BDQ-CD complex, indicating the preservation of the amorphous nature of the complex. Although there are reports about the chemical instability of drugs under accelerated conditions due to higher mobility in the amorphous state of CD drug complexes, the BDQ-CD complex developed in this study was found to be stable with a retainment of ~80% of the complexed BDQ (80.5 ± 4.6%) after 3 weeks (Figure 6B). These results also aligned well with previous reports in the literature [43,44,45].

### 2.6. Molecular Modeling

**Figure 7:**
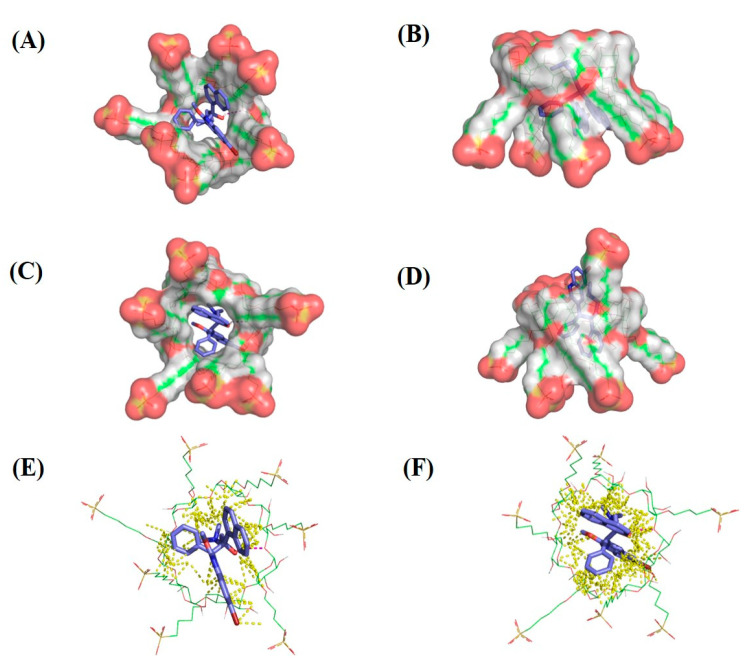
Bedaquiline docked onto the center of (**A**,**B**) SBE-β-CD-isomer 1 and (**C,D)** SBE-β-CD-isomer 2, from the perspective looking onto the top view (**A**,**C**) and side view (**B**,**D**). Hydrogen bonds are shown by dashed magenta lines and all non-polar hydrogens are hidden. Surface representations are shown for cyclodextrins. Bedaquiline docked onto the center of (**E**) SBE7β-CD-isomer 1 and (**F**) SBE7β-CD-isomer 2, from the perspective looking onto the primary face. Hydrogen bonds are shown by dashed magenta lines, yellow dashed lines show any other contacts within 3 Å radius, and all non-polar hydrogens are hidden.

Cyclodextrin molecules have a tunnel-like shape, with a lipophilic (hydrophobic) interior and a hydrophilic exterior. For drug delivery purposes, the hydrophobic interior of the cyclodextrin entraps poorly water soluble drugs allowing, for the formation of complexes [46]. Further, the addition of sulfobutyl ether groups to CD increases the length of the tunnel for drug inclusion. In this study, BDQ was docked onto SBE-β-CD isomers to examine the pose and affinity of the complex. Molecular modeling images of SBE-β-CD (Figure 7A,B) and SBE-β-CD isomer 2 (Figure 7C,D) docked with BDQ are shown in (Figure 7A,C: Top view; Figure 7B,D: Side view). BDQ appears to bind SBE-β-CD through a series of hydrophobic interactions, while a hydrophilic linker shows a potential hydrogen bonding interaction with the sulfonate group of SBE-β-CD. In both SBE-β-CD isomers, the same hydrogen bond was observed between the BDQ hydroxyl group and the cyclodextrin α-1,4-glycosidic bond oxygen, shown in Figure 7. Gold Scores ranged from 49.63 for SBE-β-CD-isomer 2 to 54.58 for SBE-β-CD-isomer 1. In Figure 7E (SBE-β-CD-isomer 1) & Figure 7F (SBE-β-CD-isomer 2), all contacts within 3Å are shown to highlight the fit of BDQ within the cyclodextrin cavity.

### 2.7. In Vitro Aerosol Performance

**Figure 8:**
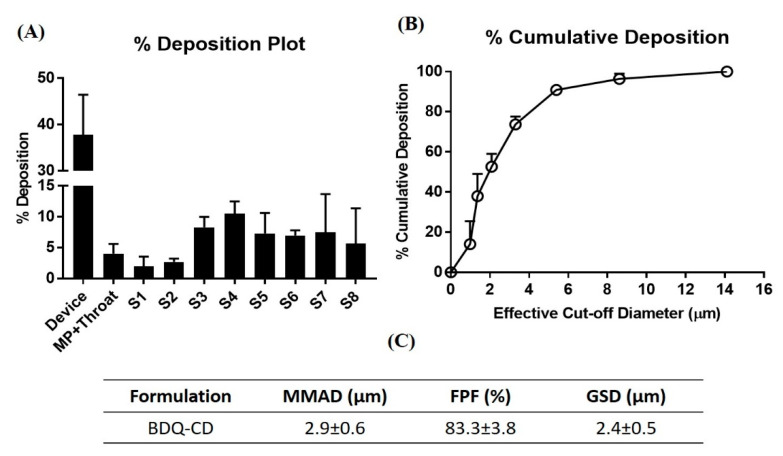
In vitro aerodynamic performance of BDQ-CD. (**A**) In vitro aerodynamic deposition of BDQ-CD in Next-Generation Impactor^TM^ (NGI) stages (**B**) % cumulative deposition of BDQ-CD in NGI. (**C**) Aerosolization properties of BDQ-CD complex. Data represent mean ± SD (*n* = 3).

Complex formation solubilizes and increases the apparent solubility of BDQ, which may further result in enhanced bioavailability of BDQ. For inhaled delivery, the use of SBE-β-CD isomers potentially offers a high lung deposition and establishes the feasibility of inclusion complex for pulmonary administration. To elaborate, cyclodextrins allow rapid dissociation of the drug from the complex, as observed in vivo studies proving the hypothesis that CDs can promote lung delivery of drugs [47]. CDs have also been evaluated as potential drug carrier in DPI formulations indicating their potential role in pulmonary drug delivery [48]. Aerosol performance of BDQ-CD inclusion complex was evaluated by assessing the aerodynamic characteristics of the particles using Next Generation Impactor (NGI), as described earlier [25]. Once nebulized, the MMAD of particles govern their deposition profile in the airways. The particle deposition (%) of BDQ-CD on each stage of the NGI, including amount deposited in mouth/throat, is plotted in Figure 8A. A large collection of particles in stage 3 and below suggests an efficient bronchi-alveolar deposition. In addition, the cumulative deposition (%) as a function of effective cut-off diameter is represented in Figure 8B, which demonstrates that 73.7 ± 3.9% of particles are within the effective cut-off diameter of 3.3 μm, which translates to stage 4 of the NGI (run at 15 L/min). Figure 8C shows the calculated aerosolization parameters of BDQ-CD: MMAD, GSD, and FPF (%). A calculated MMAD value of 2.9 ± 0.6 μm (<5 μm) was observed, which suggests that particles are within the appropriate size range for deposition into the respirable region of the lungs, while the GSD was 2.4 ± 0.5 μm, which indicates the generation of polydispersed aerosol after nebulization. It is reported that total lung deposition is greater for polydisperse aerosols compared to monodisperse aerosols, as seen in Figure 8B [49]. The FPF, also called the respirable fraction, was calculated to be 83.3 ± 3.8%, which suggests excellent aerosolization performance and that much of the dose will be deposited in the respiratory airways. The data obtained suggest that the prepared BDQ-CD inclusion complex possesses all the characteristics to render them inhalable. The aerosol characteristics of the BDQ-CD complex suggests pulmonary delivery to be a promising approach. In addition, pulmonary delivery results in minimal systemic exposure and systemic side-effects, allows for high drug concentration in the lung and frequent administration, properties ideal for an anti-cancer drug delivery. Recently, Su et al. have reported the formulation of tetrandrine-hydroxy propyl methyl cellulose complex for the treatment of bleomycin-induced pulmonary fibrosis in a murine model and observed excellent accumulation of the complex in the lungs after aerosol delivery [12]. Other studies report the ability of CDs to enhance pulmonary absorption where relative impermeability of the lungs to several drugs is posing a major obstacle after pulmonary delivery [50].

### 2.8. Cytotoxic Efficacy of BDQ and BDQ-CD Inclusion Complex in Different Cell Lines

**Figure 9:**
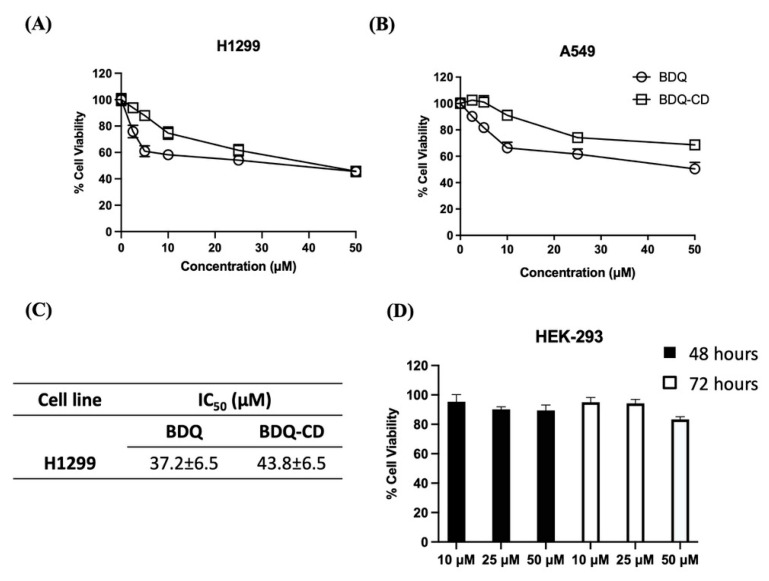
Cell viability results comparing BDQ and BDQ-CD on two different NSCLC cell lines. (**A**) H1299 and (**B**) A549. Cells were treated with different concentrations of each treatment for 72 h and cell viability was measured using MTT assay. Cells grown in media were considered as control (100%). (**C**) Table representing IC_50_ values for BDQ-CD complex. Data represent mean ± SD with *n* = 6. (**D**) Safety studies: Cytotoxicity studies on HEK-293 cell line after treatment with SBE-β-CD. Cells were incubated with equivalent amounts of SBE-β-CD to 10, 25, and 50 μM of BDQ-CD for 48 and 72 h. Data represent mean ± SD (*n* = 6).

Cell viability studies were performed using MTT assay to evaluate the cytotoxic potential of BDQ-CD complex and plain BDQ. The cytotoxic effects of BDQ and the BDQ-CD complex against two NSCLC cell lines (H1299 and A549) were quantified; and are illustrated in Figure 9A,B. For H1299 (Figure 9A) cell line, BDQ, and BDQ-CD were shown to cause cell death at higher concentration after 72 h of incubation period. As shown in Figure 9C, the IC_50_ values for BDQ and BDQ-CD complex were found to be in the similar range 37.2 ± 6.5 µM (BDQ), and 43.8 ± 6.5 µM (BDQ-CD) after 72-h incubation; whereas no IC_50_ values were observed in case of A549 cell line for both BDQ and BDQ-CD following 72-h incubation which indicates that BDQ might be effective towards specific type of NSCLC. To confirm this, further investigation will be carried out in the future to explore specificity of BDQ to exert cytotoxic potential in NSCLC cell lines. Plain BDQ, however, did not exert any significant cell death at tested concentrations, and IC_50_ values could not be determined, suggesting the need for higher doses. Similar results were observed in case of cyclodextrin inclusion complexes of erlotinib and resveratrol, as reported earlier by our research group [14,51]. In vitro studies to establish safety of optimized cyclodextrin formulation was performed on human epithelial kidney 293 cell line HEK-293. When HEK-293 cell line was treated with drug-free SBE-β-CD complex equivalent CD solution equivalent to 10, 25, and 50 μM BDQ for 48 and 72 h, cell viability of >80% following both 48- and 72-h treatment at all tested concentrations suggested that optimized BDQ-CD was not toxic to HEK-293 cells, a normal cell line. The safety profile of SBE-β-CD has been presented in Figure 9D.

## 3. Materials and Methods

### 3.1. Materials

Bedaquiline (>99%) was purchased from MedChem Express (Monmouth Junction, NJ, USA). Sulfobutyl ether-β-CD (SBE-β-CD, Dexolve^®^, average mol. wt. 1293 g/mol, average degree of substitution = 6.2–6.9 [52]) was provided as gift sample by Cyclolab (Budapest, Hungary). 3-(4,5-dimethylthiazol-2-yl)-2,5-diphenyltetrazolium bromide (MTT), dimethyl sulfoxide (DMSO), HPLC grade solvents including methanol, acetonitrile (ACN), and water were purchased from Fisher Scientific (Hampton, NH, USA).

### 3.2. Cell Lines

H1299 (Passage number #17) A549 (Passage number #23) (NSCLC) and human embryonic kidney 293 (Passage number #12) (HEK-293) cell lines were obtained from ATCC (Manassas, VA, USA). H1299 and A549 cell lines were maintained in RPMI-1640 medium (Corning Inc., Corning, NY, USA) supplemented with 10% heat-inactivated FBS (Atlanta Biologicals, Minneapolis, MN, USA), 5% sodium pyruvate (Corning Inc., Corning, NY, USA), and 5% penicillin-streptomycin (Corning Inc., Corning, NY, USA) at 5% CO_2_ and 37 °C. HEK-293 cell line was maintained in Dulbecco’s Modified Eagle’s Medium (Corning Inc., Corning, NY, USA) supplemented with 10% heat-inactivated FBS (Atlanta Biologicals, Minneapolis, MN, USA) and 5% penicillin-streptomycin (Corning Inc., Corning, NY, USA) at 5% CO_2_ and 37 °C. All cells were grown to 85–90% confluency prior to splitting or being used for experiments.

### 3.3. UV Spectrophotometric Method Development for Bedaquiline (BDQ)

A UV spectrophotometric method was developed for quantifying BDQ with a VWR Spectrophotometer (UV-1600PC; VWR, Radnor, PA, USA). Briefly, a stock solution of 1 mg/mL concentration was prepared by dissolving BDQ in acetonitrile. From the stock solution, standard solutions of 2.5, 5, 10, 20, 30, 40, 50, and 60 µg/mL were prepared by diluting with acetonitrile. All the standard solutions were scanned in the UV range 200–500 nm. In spectrum, BDQ showed maximum absorbance at 285 nm. The calibration curve was constructed between concentration and absorbance.

### 3.4. Phase Solubility Studies

Phase solubility studies of BDQ in the presence of SBE-β-CD was performed according to the method described by Higuchi and Connors [30] and previously published studies from our group with slight modifications [17,25,28]. An excess amount of BDQ (5 mg) was added to centrifuge tubes, containing different concentrations of SBE-β-CD in milliQ water (0–200 mM). Suspensions were bath sonicated for 30 min, then left at room temperature for 24 h under continuous stirring to achieve equilibrium. After 24 h, un-complexed BDQ was separated by filtration with a 0.2 μm polyvinylidene fluoride (PVDF) syringe filter. Filtered solutions were quantified for BDQ content using UV spectrophotometer at 285 nm. Phase solubility diagram was constructed by plotting the dissolved BDQ against respective concentration of SBE-β-CD. The stability constant K_c_ and complexation efficiency (CE) were calculated from phase solubility diagram using its slope and intercept values using below Equations (2) and (3), respectively.

(2) $$\mathrm{Kc}=\frac{\mathrm{Slope}}{\mathrm{So}\;\left(1-\mathrm{Slope}\right)}$$

(3) $$\mathrm{CE}=\frac{\mathrm{Slope}}{\;\left(1-\mathrm{Slope}\right)}$$

where, K_c_ is stability constant and S_o_ is the solubility of drug without cyclodextrin.

### 3.5. Continuous Variation Method (Job’s Plot Analysis)

Stoichiometry of the BDQ-CD complex was measured using the continuous variation method. In this study, Job’s plot analysis was performed to confirm the stoichiometry of BDQ with SBE-β-CD during complex formation in accordance with previously reported methods [17,28,29]. Briefly, sum of the concentrations of both components was maintained constant and the molar fraction was varied from 0.0 to 1.0. Ten mL of 1 mM solutions of both drug and cyclodextrin were prepared in methanol and water, respectively. Specifically, 1-part drug solution was mixed with 9 parts cyclodextrin solution, 2 parts of drug solution mixed with 8 parts of cyclodextrin solution, and so on. After 24 h of stirring, un-complexed BDQ was separated by filtration with 0.2 μm PVDF syringe filter and filtered solutions were analyzed by UV as described above. Stoichiometry of the complex formation was determined by plotting the change in UV absorbance × mole fraction of the drug against the mole fraction of the two components, as reported previously [17].

### 3.6. Preparation of Solid BDQ-CD Complex

The inclusion complex of solid BDQ-CD was prepared by freeze drying, as per the previously published studies from our group [25,29]. In brief, prepared BDQ-CD aqueous complex was frozen at −80 °C overnight and then placed in a freeze dryer (FreeZone 6 Liter Benchtop Freeze Dry system, LabConco, Kansas City, MO, USA) for at least 24 h. Obtained solid BDQ-CD complex was used for further solid-state characterization studies and accelerated stability studies.

### 3.7. Solid State Characterization Studies

Different techniques were used to verify the formation of BDQ-CD inclusion complex, which included ^1^H-Nuclear Magnetic Resonance Spectroscopy (^1^H-NMR), Fourier transform infrared (FT-IR) spectroscopy, Differential scanning calorimetry (DSC), and Powder X-ray diffraction (PXRD).

**^1^H-Nuclear Magnetic Resonance Spectroscopy (^1^H-NMR):**^1^H-NMR spectroscopy was used to determine chemical shift (δ) for various groups for BDQ, SBE-β-CD, physical mixture, and BDQ-CD inclusion complex, using a Bruker DPX-400 Avance (400 MHz) spectrometer (Bruker, Rheinstetten, Germany) at 300 K.

**Fourier Transform Infrared Spectroscopy (FT-IR):** FT-IR spectrum of BDQ, SBE-β-CD, physical mixture, and BDQ-CD inclusion complex were obtained using FT-IR (Perkin Elmer, Inc., Branford, CT, USA), where solvent (DMSO) was used for background correction and 10 μL of complex was used for detection. Different peaks in plotted IR spectrum were then interpreted to detect the presence of distinct BDQ groups.

**Powder X-ray Diffraction (PXRD) Studies:** X-ray diffraction spectroscopy was performed using XRD-6000 (Shimadzu, Kyoto, Japan). Diffractometer is composed of a graphite monochromator (copper-Kα1; radiation wavelength 1.5418 Å, 40 kV, and 30 mA). Samples for BDQ, SBE-β-CD, physical mixture, and BDQ-CD inclusion complex were uniformly spread on a micro-sample glass holder and then analyzed (range of 5–60°; scanning speed of 2° (2θ) /minute).

**Differential Scanning Calorimetry (DSC) Studies:** Thermograms for BDQ, SBE-β-CD, physical mixture, and BDQ-CD inclusion complex were generated using a DSC 6000 (PerkinElmer, Inc., Branford, CT, USA); equipped with an intra-cooler accessory. Briefly, a fixed amount of sample (1–5 mg) was accurately weighed, sealed in an aluminum pan, then analyzed over a temperature range (30–200 °C) and compared to a sealed empty aluminum pan maintained as reference. The heating rate was maintained at 10 °C/min under a nitrogen purge (flow rate: 50 mL/min).

### 3.8. Accelerated Stability Study

Stability of BDQ-CD complex was evaluated for 3 weeks. For this study, freeze-dried BDQ-CD powder was stored at 40 °C/75% RH. Samples were withdrawn on day 0, 7, 14, and 21, and analyzed for drug content and physicochemical stability using DSC study, as described above. Samples after week 1, 2, and 3 weeks of storage under accelerated conditions were evaluated for their thermal behavior.

### 3.9. Molecular Docking of Bedaquiline on Sulfobutyl Ether β-Cyclodextrins

Molecular docking was carried out to get further insight into how BDQ interacts with SBE-β-CD [25,29]. Implementing molecular modeling computations provides information on the orientation and interaction of BDQ and SBE-β-CD [17]. Molecular modeling studies were done on a Dell Precision workstation with Intel(R) Xeon(R) CPU E5-1620 v3 @3.50GHz processor. Energy minimization of cyclodextrin (CD) substituents were carried out in Accelrys Discovery Studio 4.1 [53]. Docking of BDQ onto CDs were performed on Genetic Optimization of Ligand Design (GOLD) docking suite 5.7.0 [17]. Docking results were visualized using PyMOL 2.3.1 [54] and Accelrys Discovery Studio.

**Structure Preparation:** The 3D structure of SBE-β-CD was extracted from the co-crystalized structure with α-amylase (PDB: 1JL8). The esters of β-CD were created in Accelrys Discovery Studio, with substitutions minimized using Drieding like force field. CD structures were saved as PDB files [55]. BDQ’s crystal structure was downloaded from The Cambridge Crystallographic Data Centre (CCDC Number: 619587) in CIF file format [56]. CIF file was opened in PyMOL and bedaquiline crystal structure was saved as a SDF file.

**Docking:** BDQ was docked onto the two SBE isomers that are of the highest occurrence in commercially available mixtures. Docking was performed to evaluate the pose and affinity of BDQ in the cyclodextrin tunnel, based on H-bonding, van der Waals interactions, metal interactions, and ligand torsion strain [57]. The docking parameters used include the chemscore kinase template, a centroid point to define the binding site, standard ligand flexibility, GOLD Score as a scoring function, and 200% search efficiency. A higher GOLD Score is indicative of higher affinity between the ligand and receptor.

### 3.10. In Vitro Aerosol Performance

According to previously published studies, Next Generation Impactor™ (M170 NGI: MSP Corporation, Shoreview, MN, USA) was used to evaluate In vitro lung deposition of BDQ-CD inclusion complex [27]. NGI separates particles based on their aerodynamic size which is useful in predicting *in-vivo* deposition of particles [58]. The NGI was equipped with a stainless-steel induction port (USP throat adaptor) attachment and specialized stainless-steel NGI™ gravimetric insert cups (NGI Model 170, MSP Corporation, Shoreview, MN, USA). Prior to operation, the NGI plates were placed in refrigerator (4 °C; 90 min) for pre-conditioning to minimize solvent evaporation during the process. To test In vitro lung deposition, 2 mL of BDQ-CD (BDQ-CD diluted 2-fold; CD concentration: 0.13 g/mL) formulation was placed into a PARI LC PLUS^®^ nebulizer cup of Pari FAST-NEB compressor system. (Boehringer Ingelheim, Inc. Ridgefield, CT, USA) attached to a customized rubber mouthpiece, connected to the NGI. A Copley HCP5 vacuum pump (Copley Scientific, Nottingham, UK) was used to attain a flow rate of 15 L/min with flow rate adjusted using a Copley DFM 2000 flow meter (Copley Scientific, Nottingham, UK). The formulation was run for a total of 4 min with 30 s nebulizer priming. After the run, samples were collected from every stage (1–8), as well as from throat and induction port using ACN: Water (50:50) for rinsing. Samples were then analyzed using previously established UV method. Drug content and effective deposition was calculated for dose deposited at each stage. Mass median aerodynamic diameter (MMAD) and fine particle fraction (FPF) of inhaled therapeutics determines their fate and the site of deposition in lungs [59]. MMAD within the range of 1–5 µm is essential for accumulation of therapeutics in the respirable airways. To give an indication of aerosolization performance, fraction of the emitted dose deposited in the NGI stages with d_ae_ < 5.39 μm (Fine particle fraction; FPF %) was calculated. To determine the spread of aerodynamic particle size distribution, mass median aerodynamic diameter (MMAD, d_ae_ < 5.00 μm) and the geometric standard deviation (GSD) were calculated, as reported earlier [27,60]. Experiments were done in triplicate (*n* = 3) and data are represented as mean ± SD.

### 3.11. Cytotoxicity Assay

Plain BDQ and prepared BDQ-CD inclusion complex (200 mM SBE-β-CD) were evaluated for their cytotoxic activity in two different NSCLC cancer cell lines (H1299 and A549). The BDQ complex with 200 mM SBE-β-CD was evaluated for its cytotoxic effect in this study, as SBE-β-CD resulted in superior solubilization of BDQ and exhibited a stoichiometric ratio of 1:1 (Discussed in Section 2.2). The experiment was carried out using an MTT assay, as per established protocols utilized in our lab [61,62,63]. In brief, cells were seeded in tissue culture (TC) treated 96-well plates at a seeding density of 2500 cells/well, incubated overnight for adherence at 37 °C and 5% CO_2_, and treatments were added the next day at various concentrations (2.5, 5, 10, 25, and 50 µM) of BDQ and BDQ-CD. Equivalent amounts of freeze-dried samples of BDQ-CD were used in this study in place of cyclodextrin solution. After incubating for 72 h, the media was aspirated from the wells, followed by a 2-h incubation with MTT solution. The MTT solution was then aspirated and 100 µL of DMSO was added to each well. The plates were then kept for shaking for 30 min and the absorbance was read on a Spark 10 M (Tecan, Männedorf, Switzerland) plate reader at 570 nm. Safety of CD complex formulation was determined by evaluating the cytotoxicity of SBE-β-CD on healthy non-cancerous human embryonic kidney 293 cells (HEK-293). Briefly, cells were seeded into 96-well plates as described earlier and were incubated overnight followed by treatment with equivalent amounts of CD solutions corresponding to 10, 25, and 50 μM of BDQ-CD for 48 and 72 h. % cell viabilities were determined through MTT assay, as described earlier.

## 4. Conclusions

This study focused on improving the solubility of BDQ to enhance its biological efficacy against non-small cell lung cancer, through complexation with cyclodextrins. The current study highlighted BDQ’s repurposing potential as an anti-cancer drug, presenting a promising strategy in the new drug development for cancer. A ≈ 2.8×10^3^-fold solubility enhancement was achieved for BDQ with complexation using SBE-β-CD. Further, owing to the improved aqueous solubility, the BDQ-CD complex showed a slightly enhanced cytotoxic effect on H1299 NSCLC cancer cell as BDQ (free drug). In addition, the aerosol properties of the complex demonstrate inhalation as a promising strategy for the delivery of BDQ. Taken together, these results validate the hypothesis of this study, while providing novelty by predicting the pulmonary delivery potential for inhalation therapy and superior efficacy of the BDQ-CD inclusion complex in lung cancer cell lines.

## Data Availability

The data shown in this study are presented in the article. Also, the data presented in this study are available on request from the corresponding authors.
